# Isolation and Cultivation of Porcine Endothelial Cells, Pericytes and Astrocytes to Develop an In Vitro Blood–Brain Barrier Model for Drug Permeation Testing

**DOI:** 10.3390/pharmaceutics15061688

**Published:** 2023-06-08

**Authors:** Verena Ledwig, Stephan Reichl

**Affiliations:** 1 Center of Pharmaceutical Engineering (PVZ), Technische Universität Braunschweig, Franz-Liszt-Straße 35A, 38106 Braunschweig, Germany; v.ledwig@tu-braunschweig.de; 2 Institute of Pharmaceutical Technology and Biopharmaceutics, Technische Universität Braunschweig, Mendelssohnstraße 1, 38106 Braunschweig, Germany

**Keywords:** blood–brain barrier, endothelial cells, pericytes, astrocytes, coculture, in vitro model, drug permeation, transendothelial electrical resistance

## Abstract

The blood–brain barrier (BBB) is the bottleneck in the development of new drugs to reach the brain. Due to the BBB, toxic substances cannot enter the brain, but promising drug candidates also pass the BBB poorly. Suitable in vitro BBB models are therefore of particular importance during the preclinical development process, as they can not only reduce animal testing but also enable new drugs to be developed more quickly. The aim of this study was to isolate cerebral endothelial cells, pericytes, and astrocytes from the porcine brain to produce a primary model of the BBB. Additionally, as primary cells are well suited by their properties but the isolation is complex and better reproducibility with immortalized cells must be ensured, there is a high demand for immortalized cells with suitable properties for use as a BBB model. Thus, isolated primary cells can also serve as the basis for a suitable immortalization technique to generate new cell lines. In this work, cerebral endothelial cells, pericytes, and astrocytes were successfully isolated and expanded using a mechanical/enzymatic method. Furthermore, in a triple coculture model, the cells showed a significant increase in barrier integrity compared with endothelial cell monoculture, as determined by transendothelial electrical resistance measurement and permeation studies using sodium fluorescein. The results demonstrate the opportunity to obtain all three cell types significantly involved in BBB formation from one species, thus providing a suitable tool for testing the permeation properties of new drug candidates. In addition, the protocols are a promising starting point to generate new cell lines of BBB-forming cells as a novel approach for BBB in vitro models.

## 1. Introduction

The BBB is a physiologic barrier between the bloodstream and the central nervous system. This barrier is mainly formed by endothelial cells of cerebral microvessels under the influence of connected neighboring cells, such as pericytes and astrocytes, and it serves as a highly selective filter to maintain the homeostasis of the brain. Due to the characteristics of the BBB, most of the newly developed active compounds against cerebral diseases cannot penetrate into the central nervous system [1]. For testing new drug candidates, such as investigating their transport across the BBB, in vitro BBB models are a promising tool as a replacement for animal models. Various in vitro models have already been described, ranging from endothelial cell monoculture models [2,3,4] to coculture models in direct and indirect contact with endothelial cells, pericytes, astrocytes, and partly also neurons [5,6,7]. Monoculture models are highly simplified because they do not represent cell-to-cell communication between different cell types. It has been shown that endothelial cells are influenced in their properties by the surrounding cells [8]. Thus, cell–cell contacts and interactions are necessary for the maintenance of endothelial cell barrier integrity (for review see [9,10]). More organotypic triple coculture models have been previously shown to have higher barrier integrity because of the interaction of pericytes and astrocytes with endothelial cells [11,12]. Not only the increased barrier of the endothelial cells but also the organotypic structure of the cell assembly argues for a triple-coculture model. The origin of the cells of the individual models described in the literature is very heterogeneous. Most models are based on cells from rats and mice due to their availability and cost, but there are also models from other species, such as pigs and bovines. Human BBB models have been generated mainly with immortalized cells due to the lack of availability of primary cells and for ethical reasons. Recently, induced pluripotent stem cells have been increasingly used and show great potential (for review see [6,8,13,14]).

Primary cells offer the advantage of organotypicity. Among other things, they express organotypic transport proteins and connecting proteins for cell–cell contact, thus providing functional properties in vitro comparable with those in vivo. However, limited lifetime and poor reproducibility are the major drawbacks of using primary cells. The use of immortalized cells is supported by good reproducibility and easy handling, but the functional properties are often not comparable with the in vivo situation. Additionally, because the immortalized endothelial cells described in the literature, such as the commonly used hCMEC/D3 cell line, form only a weak barrier, these models are not well suited for permeation models [15,16]. As there is currently no good BBB permeation model using immortalized cells, yet there is a high need for preclinical testing, new approaches for the development of such in vitro drug testing models are of particular importance.

Lipps et al. described a new technology that can be used to generate immortalized cells that are functionally and phenotypically comparable with primary cells [17]. The aim of this work was therefore to obtain a valid cultivation protocol for primary cells as a basis for subsequent immortalization according to Lipps et al. to ultimately generate a functional immortalized BBB model. As a basis, it is important to first establish a good BBB model with primary cells to transfer the techniques to the later immortalized model.

Primary porcine cells were used in this work because porcine endothelial cells are well characterized with respect to their transporter expression and junctional proteins. Another advantage is that primary porcine cells are highly available, as porcine brains are a waste product of the slaughter process. Because there are some similarities between porcine and human vascular physiology and because the cell yield of endothelial cells per porcine brain is high (approximately 50 million endothelial cells), the porcine model is very interesting for high-throughput drug screening [8].

Consequently, the aim of this work was therefore to isolate all three cell types in one isolation procedure and thus to create a suitable permeation model. The focus was on the optimization of the isolation method and the investigation of different approaches of triple cocultures in direct comparison to investigate the effects of different arrangements of the cells to each other as well as the order of seeding. The idea is to generate functional cell material as a basis for subsequent immortalization that is suitable as an in vitro coculture model of the BBB. On the one hand, the isolated cells can be used as a primary blood–brain barrier model of a species; on the other hand, the isolated primary cells can also be used as a basis for subsequent new immortalization approaches, thus offering the possibility to study the primary and immortalized models in direct comparison.

## 2. Materials and Methods

### 2.1. Materials

Cell culture flasks and 8-well chamber slides were obtained from Sarstedt (Nümbrecht, Germany). ThinCert^®^ inserts were purchased from Greiner Bio-One (Bad Nenndorf, Germany). Twelve-well plates were obtained from Corning Costar (Kennebunk, ME, USA). Phosphate-buffered saline (PBS), collagen G, human collagen IV, human laminin, human fibronectin, poly-L-lysine, dispase, dextran and puromycin were all received from Sigma–Aldrich (Munich, Germany). Collagenase/dispase was obtained from Roche (Basel, Switzerland). A 150 μm polyamid mesh was obtained from Biologie Bedarf Thorns (Deggendorf, Germany). Percoll (1.131 g/mL) was purchased from GE HealthCare (Chicago, IL, USA). The media Earle’s Medium 199 (M199) (FG0615), Dulbecco’s modified Eagle’s medium (DMEM) (F0445) and DMEM/F–12 (1:1) (F4815) and fetal bovine serum (FBS), horse serum, hydrocortisone (HC), HEPES and gentamycin (10 mg/mL) were acquired from Biochrom (Berlin, Germany). Polysorbate 20 and Eppendorf tubes were all purchased from Carl Roth (Karlsruhe, Germany). Sucrose, 8-(4-chlorophenylthio) adenosine 3′,5′-cyclic monophosphate sodium salt (pCPT-cAMP), antibiotic–antimycotic solution (penicillin 10,000 U/mL, streptomycin 10 mg/mL, and amphotericin B 25 μg/mL), paraformaldehyde (PFA), sodium fluorescein and Hoechst 33342 (2′-[4-ethoxyphenyl]-5-[4-methyl-1-piperazinyl]-2,5′-bi-1H-benzimidazol-trihydrochlorid-trihydrate) were received from Sigma–Aldrich (Munich, Germany). Krebs-Ringer buffer (KRB) contained 6.8 g NaCl, 0.4 g KCl, 0.14 g NaH_2_PO_4_·H_2_O, 2.1 g NaHCO_3_, 3.575 g HEPES, 1.1 g D-glucose monohydrate, 0.2 g MgSO_4_·7 H_2_O and 0.26 g CaCl_2_·2 H_2_O in 1000 mL of double-distilled water. L-glutamine and the phosphodiesterase 4 inhibitor Ro-20–1724 were purchased from Merck (Darmstadt, Germany). Dimethyl sulfoxide (DMSO) and trypsin–EDTA (0.5/0.2 g/L) were obtained from Thermo Fisher Scientific (Waltham, MA, USA). Triton X-100 was received from ICN Biomedicals Inc. (Irvine, CA, USA). Antibodies for immunofluorescence, rabbit anti-von Willebrand factor (vWF) (ab6994), mouse anti-alpha-smooth muscle actin (SMA) (ab7817), mouse-anti glial fibrillary acidic protein (GFAP) (ab190288), rabbit anti-claudin 5 (ab15106), rabbit anti-occludin (ab31721), rabbit anti-VE-cadherin (ab33168) and fluorescence-labeled secondary antibody goat anti-rabbit IgG (ab97079) and goat anti-mouse IgG (ab175473) were all obtained from Abcam (Cambridge, UK). Normal goat serum was acquired from BIOZOL (Eching, Germany).

### 2.2. Cell Isolation

**Figure 1:**
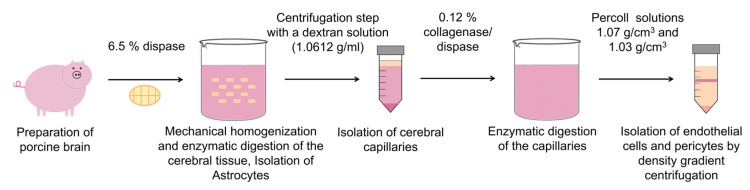
Flow scheme of the isolation method.

Using the method described here, all three cell types that are significantly involved in the formation of the blood–brain barrier can be isolated from one organ. A flow scheme of the isolation protocol is illustrated in Figure 1.

#### 2.2.1. Endothelial Cells

Primary cultures of porcine brain endothelial cells (PBECs) were isolated based on the methods of Bowman et al. [18] and Franke et al. [19] with our own modifications. Brains were obtained from 6-month-old pigs from a nearby slaughterhouse. As the brains were a waste product of the slaughtering process, no animals were killed for this cell isolation. For one batch, 10 intact hemispheres were needed. An initial quality control was conducted directly when the hemispheres were removed from the halved pigs. Only intact hemispheres were used to reduce the risk of contamination with epithelial cells. After removal of the hemispheres, a washing step with 70% ethanol was performed to prevent bacterial contamination. The hemispheres were then rinsed with cold PBS and transported to the laboratory in fresh cold PBS with 2% antibiotic–antimycotic solution in a round sealable transport box on ice. One to two hours after removal of the brains, further processing occurred in the laboratory. After another washing step with PBS, the 10 hemispheres were transferred into a sterile 2 liter beaker with 500 mL PBS supplemented with 2% antibiotic–antimycotic solution. The next steps were performed under laminar air flow conditions. Next, the meninges and secretory brain areas were carefully removed, and the remaining cerebral tissue was mechanically homogenized. The homogenized brain was transferred into prewarmed preparation medium (M199 supplemented with 1% antibiotic–antimycotic solution, 0.1 mg/mL gentamycin and 10 mM HEPES buffer). After 2 h enzymatic digestion at 37 °C with 6.5% dispase, a centrifugation step followed (6800× *g*, 10 min at 4 °C) with a dextran solution (1.0612 g/mL). The isolated cerebral capillaries were further triturated and filtered through a 150 μm polyamide mesh before a second enzymatic digestion step with a collagenase/dispase solution (1.22 mg/mL). The released endothelial cells were collected from the interface of a density gradient centrifugation step by using two different densities of Percoll solutions (1.07 g/cm^3^, 1.03 g/cm^3^, 104× *g*, 10 min). Primary cerebral endothelial cells were washed once with the preparation medium before seeding onto 9 collagen-coated plastic flasks (each with 175 cm^2^ growth area).

The endothelial cells were seeded in plating medium (M199 supplemented with 1% antibiotic–antimycotic solution, 10% horse serum and 0.1 mg/mL gentamycin) containing 2 μg/mL puromycin [20] to reduce contaminating cells, such as pericytes. For coating, collagen G was diluted 1:30 with demineralized water, and the surface of the culture vessels was wetted with the prepared solution. The culture vessels were placed under a laminar air flow until complete evaporation of the liquid.

After 24 h of cultivation, cells were washed with PBS^++^ (containing 0.5 mM MgCl_2_ and 0.9 mM CaCl_2_) and cultivated with fresh cultivation medium (M199 supplemented with 1% antibiotic–antimycotic solution and 10% horse serum) containing 2.2 μg/mL puromycin for an additional 24 h. When the cells reached 90% confluency, they were cryopreserved in liquid nitrogen for later use.

#### 2.2.2. Pericytes

Porcine cerebral pericytes were obtained simultaneously with the same isolation procedure as the PBECs, and only the conditions of cultivation differed. The growth-supporting conditions for pericytes comprised cultivation on uncoated dishes with DMEM supplemented with 10% FCS, 1% antibiotic–antimycotic solution, 0.1 mg/mL gentamycin and 4 mM L-glutamine (pericytes-astrocytes culture medium). The cells were cultured until the 2nd passage and then cryopreserved for later use.

#### 2.2.3. Astrocytes

Porcine cerebral astrocytes were obtained after the first enzymatic digestion step with 6.5% dispase. Here, 5% of the cell suspension was collected and diluted 1:5 with pericytes-astrocytes culture medium. After a centrifugation step (1000 rpm, 5 min), the pellet was resuspended in pericytes–astrocytes culture medium, and the cells were plated on poly-L-lysine-coated dishes. The coating was made shortly before according to the manufacturer’s specifications. The cells were cultured until the 2nd passage and then cryopreserved in liquid nitrogen for later use.

### 2.3. Construction of the BBB Model

Four different coculture models were studied in comparison with the endothelial monoculture to obtain the best effect of the coculture regarding the barrier integrity. PBECs were seeded (2.21 × 10^5^ cells/cm^2^) on the apical side of the coated (collagen IV, fibronectin, and laminin (5:2:3), 25 μg/mL each) ThinCert^®^ insert membrane (polyethylene terephthalate, 0.4 μm pore size) or in direct contact with the pericytes.

**Figure 2:**
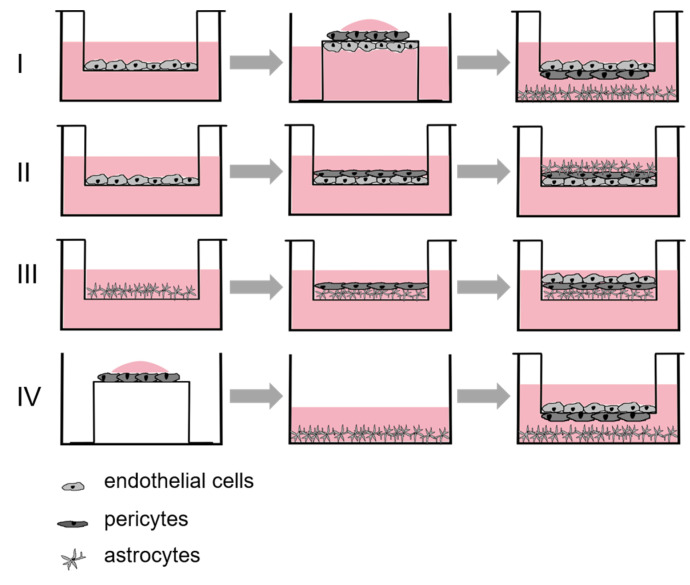
Coculture models. (**I**): First, endothelial cells were seeded, and the next day, pericytes and astrocytes were seeded. Direct coculture of all three cell types in different seeding order. (**II**): Endothelial cells–pericytes–astrocytes. (**III**): Astrocytes–pericytes–endothelial cells. (**IV**): Pericytes and astrocytes were seeded first, and endothelial cells were seeded the next day.

Pericytes (1.5 × 10^4^ cells/cm^2^) were seeded in direct contact with endothelial cells or on the basolateral side of the insert membrane, and astrocytes (1.5 × 10^4^ cells/cm^2^) were seeded on the poly-L-lysine-coated 12-well bottom or in direct contact with the pericytes. The coculture medium used consisted of DMEM/F12 (1:1) medium with the addition of L-glutamine (4 mM), 10% FBS, 1% antibiotic–antimycotic solution, and 0.1 mg/mL gentamycin. In coculture model I (see Figure 2), PBECs were seeded first, and pericytes and astrocytes were seeded the next day in indirect coculture. In coculture models II and III, all three cell types were seeded on top of each other in direct contact but in different orders. In coculture models I and II, PBECs were allowed to adhere overnight, pericytes were seeded the next day, and then astrocytes were seeded in a 3 h offset. The direct seeding of cells in coculture model III was performed the other way in reverse. Here, astrocytes were seeded first, followed by pericytes in a 3 h offset, and finally, PBECs were seeded the next day. In coculture model IV, the astrocytes were first seeded on the well bottom, and the pericytes were seeded on the basolateral membrane side of the insert. The next day, PBECs were seeded on the inserts for indirect coculture. After two days of cocultivation of the four different coculture models with coculture medium, a medium change to a differentiation medium was performed to induce an increase in barrier integrity, as indicated by higher transendothelial electrical resistance (TEER) values. The differentiation medium used consisted of DMEM/F12 (1:1) medium with the addition of L-glutamine (4 mM), 1% antibiotic– antimycotic solution and hydrocortisone (0.55 μM, for coculture I and III) [21], and additional pCPT-cAMP (250 μM) and RO-20-1724 (17.5 μM) [22] for cocultures II and IV.

### 2.4. Immunostaining

For immunofluorescence staining, the cells to be examined were seeded on 8-well chamber slides and cultured to 80% confluence. When junctional proteins were evaluated in the PBECs, the cells were cultured to complete confluence. Cells were fixed with 0.4% PFA solution in PBS with 10% sucrose for 10 min. Subsequently, the cells were permeabilized with 0.1% Triton X-100 in PBS. Nonspecific binding was saturated with 10% normal goat serum in PBST (PBS containing 0.1% polysorbate 20) for 45 min at room temperature. Incubation with the specific primary antibody was performed at 4 °C overnight (anti-vWF, anti-VE-cadherin, anti-occludin, anti-claudin-5, anti-alpha-SMA and anti-GFAP). This was followed by incubation with the fluorescence-labeled secondary antibody (1 h at room temperature). Alexa Fluor 568-conjugated anti-mouse immunoglobulin and FITC-conjugated anti-rabbit immunoglobulin were used as secondary antibodies. Cell nuclei were counterstained with Hoechst 33342 (20 μg/mL) in PBS for 15 min at room temperature.

To determine the purity of the isolated cells, immunofluorescence staining of the cell-specific proteins vWF, alpha-SMA, and GFAP was performed in each cell population to determine the respective proportions of endothelial cells, pericytes, and astrocytes. Next, 10 fluorescence images of at least 3 wells were randomly acquired using a fluorescence microscope (Olympus, Hamburg, Germany) equipped with cell F software (Olympus) and finally analyzed using the ImageJ program (U. S. National Institutes of Health, Bethesda, MD, USA). The counted nuclei were set to 100%, and the specifically stained cells were set in proportion to the nuclei.

### 2.5. Transendothelial Electrical Resistance Measurements

The barrier properties of the cerebral endothelial cells were assessed by TEER measurements using an Endohm chamber and EVOM resistance meter (World Precision Instruments, Sarasota, FL, USA). To create comparability of the various in vitro BBB coculture models, the TEER value was normalized to the endothelial cell monoculture in each case to consider only the effect of the coculture on barrier integrity.

### 2.6. Permeation Studies

To investigate BBB permeability, an absorption study was performed using sodium fluorescein, which is a hydrophilic marker molecule for paracellular drug transport. The donor solution (250 μg/mL sodium fluorescein in KRB) was pipetted onto the endothelial cells of the triple coculture, and after 10, 20, 30, 50, 70, 100, 126, 255, and 350 min, 100 μL of sample was withdrawn from the acceptor and replaced by fresh KRB. The samples were analyzed using a multiplate reader from Tecan (Männedorf, Switzerland) with excitation wavelengths of 485 nm and emission wavelengths of 535 nm. The apparent permeation coefficient was determined as described previously [23].

### 2.7. Statistics

The experiments were performed at least in triplicate. All results are presented as the mean ± SD. To compare TEER values between the respective coculture and monoculture, a two-sample *t*-test was performed for each variant (using the program OriginPro 2019; OriginLab, Northampton, MA, USA). A two-way ANOVA followed by Bonferroni post hoc tests was also performed to compare the coculture models (using GraphPad Prism version 9.5.1; GraphPad Software, San Diego, CA, USA). *p*-values less than 0.05 were considered statistically significant.

## 3. Results

### 3.1. Characterization of the Isolated Cells

**Figure 3:**
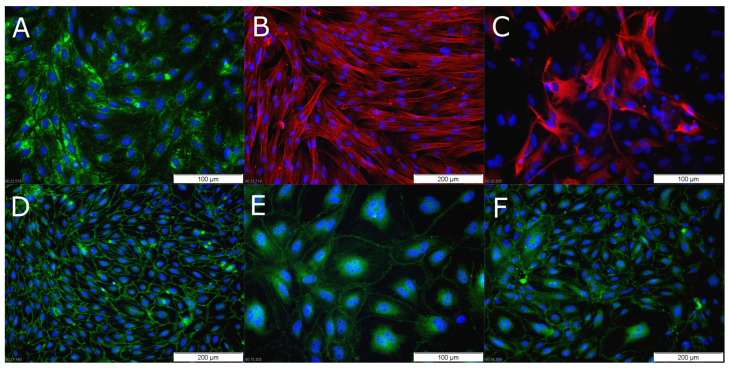
Immunofluorescence staining of cerebral porcine microvascular endothelial cells ((**A**): vWF, (**D**): VE-cadherin, (**E**): claudin-5, and (**F**): occludin), pericytes ((**B**): alpha-SMA) and astrocytes ((**C**): GFAP). Nuclear staining: Hoechst.

All three cell types could be isolated and cultivated using the method described here. Endothelial cells grew confluent within 2–3 days after preparation and could either be used directly for experiments or initially cryopreserved. Because pericytes and astrocytes were cultivated to passage 2 (to achieve higher purity, data not shown), the culturing of cells after preparation took 10–22 days for pericytes and 22–30 days for astrocytes to achieve complete confluence in passage 2. Endothelial cells were used at passage 1, pericytes were used at passage 3 to passage 7, and astrocytes were used at passage 3 to passage 4 without any change in morphology or growth behavior. The cell populations were characterized by immunofluorescence staining of specific marker proteins. As shown in Figure 3, PBECs were identified by the endothelial-specific protein vWF, pericytes were identified by alpha-SMA and astrocytes were identified by GFAP. The expression of organotypic proteins, which are essential for the development of the BBB, was also investigated in PBECs. PBECs express junctional proteins such as VE-cadherin, claudin-5, and occludin (see Figure 3).

### 3.2. Purity of Isolated Cells

**Figure 4:**
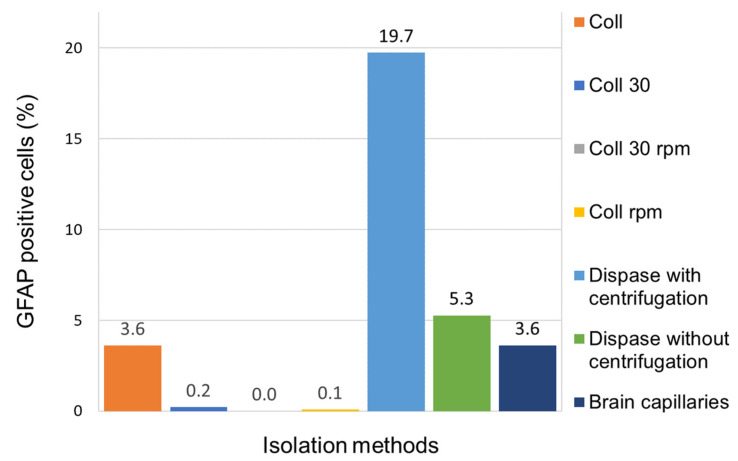
Isolation methods to purify the astrocytes and the number of GFAP-positive cells.

PBECs could be isolated with a high purity of 99.9%, and only 0.1% of the cells were detected as pericytes. The pericytes were also isolated with high purity (100%), and the astrocytes were obtained with a purity of 20%, with the remaining 80% identified as pericytes. However, although astrocytes are a mixture of cells with pericytes, the cells will be referred to only as astrocytes hereafter because the effect of astrocytes in the coculture experiments is of interest. Figure 4 shows the number of GFAP-positive cells (in %) of the different isolation techniques. An initial attempt was made to increase the purity of astrocytes from the cell pool after the second enzymatic digestion step of the cell isolation protocol (see Section 2.2.1) with collagenase/dispase (Figure 4, coll). Bobilya et al. postulated that astrocytes adhere faster (during 30 min) than other cell types and thus can be purified in a cell mixture [24]. This approach was tested, and a medium change was performed after 30 min to remove contaminating cells such as pericytes (see Figure 4, Coll 30). An additional approach was tested, which is based on a shaking step to shake off contaminating cells [25,26,27]. The cells were shaken at 80% confluence for 18 h at 220 rpm at 37 °C [27]. This method was tested on the cell pool (coll) as well as on potentially already purified cells after a 30 min adhesion time (coll 30). However, no increased astrocyte population was observed in any of the purification steps (coll 30, coll 30 rpm, and coll rpm) compared with the starting cell pool (coll). Likewise, no increased number of astrocytes could be isolated using an outgrowth method starting from isolated brain capillaries (Figure 4, brain capillaries). Furthermore, astrocyte purity was tested after the first enzymatic digestion of the brain with dispase with and without an additional centrifugation step prior to plating. Here, the highest degree of astrocyte purity (19.7%) was observed for the method with the additional centrifugation step (for details, see Section 2.2.3). The isolation method with the highest purity of astrocytes was used in the following for the generation of a BBB model.

### 3.3. Static Primary Coculture Model

#### 3.3.1. Influence of the Seeding Orientation of the Triple Coculture on the Barrier Integrity

**Figure 5:**
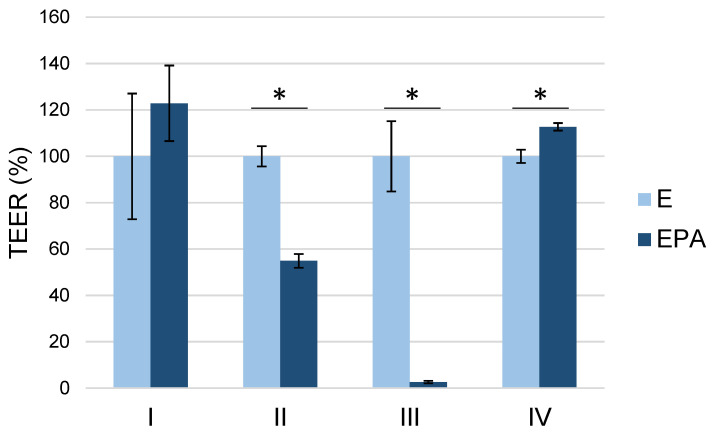
Influence of different seeding orders and orientations of the triple coculture (EPA) on the TEER compared with the endothelial cell monoculture (E). The data were normalized to the endothelial cell monoculture; n = 3–4, mean ± SD; * *p* < 0.05.

To ensure the best possible influence of triple coculture on the barrier integrity of endothelial cells, different seeding procedures and orientations of endothelial cells, pericytes and astrocytes were tested. The TEER values obtained are displayed in Figure 5. While the TEER values are partly quite different, in this study, we only wanted to investigate the influence of the coculture versus the endothelial cell monoculture; thus, the data were normalized to the respective endothelial cell monoculture for better comparison. Direct seeding of all three cell types on top of each other in different orders (cocultures II and III) resulted in TEER value reduction in each case compared with endothelial cells in monoculture. A possible explanation for this effect could be the reorientation of endothelial cells to capillary-like structures, which we observed in an immunohistochemical study (data not shown). Cocultures I and IV each showed a positive effect of the triple coculture compared with the endothelial cell monoculture in terms of increased TEER value, whereas only coculture IV could reach a significant increase in TEER value (two-sample *t*-test, *p*-value < 0.05). The high standard deviations in coculture model I are probably because the endothelial cells were exposed to hydrostatic pressure by upside-down cultivation when seeding the pericytes and astrocytes. This cultivation method was apparently not tolerated by the endothelial cells as well as coculture model IV. For the comparison of the cocultures among each other, a two-way ANOVA with Bonferroni post hoc tests was also performed. Again, a significant positive effect of coculture could only be found for coculture variant IV (*p*-value < 0.001). Thus, coculture model IV emerged as the best model, which was used for coculture experiments in the following.

#### 3.3.2. Permeation Studies

**Figure 6:**
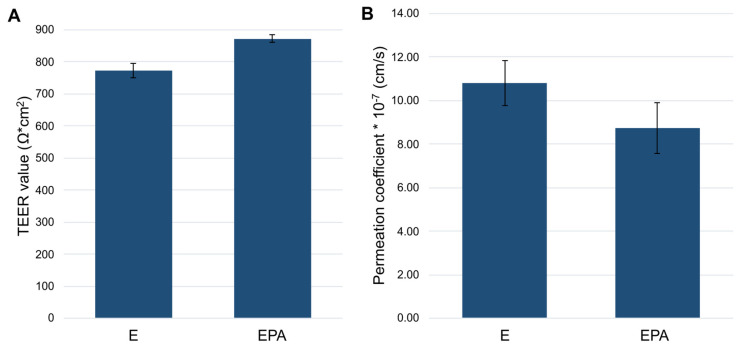
Comparison of the TEER values (**A**) and the permeation coefficients of sodium fluorescein (**B**) of the triple coculture (EPA) compared with the endothelial cells in monoculture (E). n = 3, mean ± SD.

The triple coculture model with the best effect on barrier integrity (coculture model IV) was subsequently tested with a hydrophilic marker substance (sodium fluorescein, 250 μg/mL) with regard to permeation properties. Figure 6 provides a comparison of the TEER values and the achieved permeation coefficients (P_app_) for both the triple coculture model (EPA) and the endothelial cells in monoculture (E). In the case of the triple coculture model, a lower P_app_ value was detected than for the endothelial monolayer (E: 1.08 × 10^−6^ ± 1.05 × 10^−7^ cm/s; EPA: 8.72 × 10^−7^ ± 1.17 × 10^−7^ cm/s). These data were correlated with the TEER value data obtained and suggest that increases in endothelial cell barrier integrity were induced by coculture with pericytes and astrocytes (E: 733 ± 22 Ω·cm^2^, EPA: 872 ± 12 Ω·cm^2^).

## 4. Discussion

The protocols reported here allowed microvascular endothelial cells, astrocytes, and pericytes to be isolated and cultured within one preparation step from the porcine brain. Organotypic junction proteins were detected in endothelial cells (occludin, claudin-5, and VE-cadherin). VE-cadherin supports cell–cell junction stabilization [28,29], and claudin-5 and occludin are considered to be key elements in the expression of cellular barrier function [30,31]. Although astrocyte cultures could not be obtained purely, the triple coculture protocol generated a BBB model with high TEER (approx. 900 Ω·cm^2^) and low sodium fluorescein permeability (P_app_ approx. 8.5 × 10^−7^ cm/s). This inverse behavior between P_app_ and the TEER value has already been reported for an in vitro BBB model by Gaillard and de Boer [32]. The TEER values gained with triple coculture model IV were in a similar range as described before for BBB models with sufficient barrier characteristics [22,33]. Patabendige et al. reported a permeation coefficient of P_app_~1 × 10^−6^ cm/s for mannitol using PBECs and TEER values of 700–800 Ω·cm^2^ [22]. In contrast to our primary cell culture model, BBB models based on the well-established human cell line hCMEC/D3 generally do not express such a high endothelial barrier, resulting in considerably lower TEER and higher P_app_ values for hydrophilic compounds (e.g., sodium fluorescein), as described by Hinkel and coworkers (TEER: 40 Ω·cm^2^, P_app_: 5 × 10^−6^ cm/s; [16]). However, the expression of cellular barrier integrity is considered to be essential for a valid in vitro BBB model and has a high degree of organotypicity due to cell–cell interactions during cultivation. In this context, it has already been shown that coculture of endothelial cells with astrocytes is necessary in the development of an in vivo adapted BBB model for preclinical drug testing to achieve not only an appropriate diffusion barrier but also similar expression patterns of transport proteins and enzymes [33,34]. Thus, the model presented here is a good basis for further investigations regarding the generation of new cell lines [17] and comparison with the primary model. However, if the cells are to be immortalized in the following, purity must be considered. As the PBECs and the pericytes could be isolated very purely, this concerns only the astrocytes. One approach is to purify astrocytes after immortalization. For this purpose, astrocytes could be immortalized first, and then individual clones could be selected, characterized and expanded further. This method of harvesting single cells and their subsequent expansion has already been successfully performed in our laboratory with primary astrocytes. In fact, a pure astrocyte population could be generated, but the cells could not be expanded to an appropriate cell number. 

## 5. Conclusions

The study showed that all three cell types of the BBB can be isolated and expanded as primary cultures. By coculture, a model of the blood–brain barrier could be constructed that exhibits high barrier properties and low permeability. The cells thus represent a good starting position for the subsequent immortalization step. However, further studies are needed to demonstrate whether pure cultures of the cell lines, especially astrocytes, can be established and whether these cell lines can also be used to create a BBB model with comparable barrier characteristics. In addition, it must be shown whether this approach can be transferred to human cells.

## Data Availability

Data available upon reasonable request from corresponding author.
